# P-1124. Implementation and Evaluation of a Pediatric Tele-Antimicrobial Stewardship Program in an Integrated Healthcare System

**DOI:** 10.1093/ofid/ofae631.1311

**Published:** 2025-01-29

**Authors:** Jena Stallsmith, Jared Olson, Adam Hersh, Marilyn E Valentine, Emily A Thorell

**Affiliations:** Primary Children's Medical Center, Salt Lake City, Utah; Primary Children's Hospital, Salt Lake City, Utah; University of Utah, Salt Lake City, UT; University of Utah, Salt Lake City, UT; University of Utah, Salt Lake City, UT

## Abstract

**Background:**

Intermountain Health (IH) has had a successful adult telehealth antimicrobial stewardship program (ASP) since 2016, and Primary Children’s Hospital has also had a longstanding ASP. With 298 pediatric beds throughout the other 23 hospitals within IH, there was an opportunity to expand to these patients that were not previously reviewed. The purpose of this study is to describe the implementation and outcomes of a pediatric tele-antimicrobial stewardship program (PT-ASP) in these hospitals throughout an integrated healthcare system.

**Figure 1:**
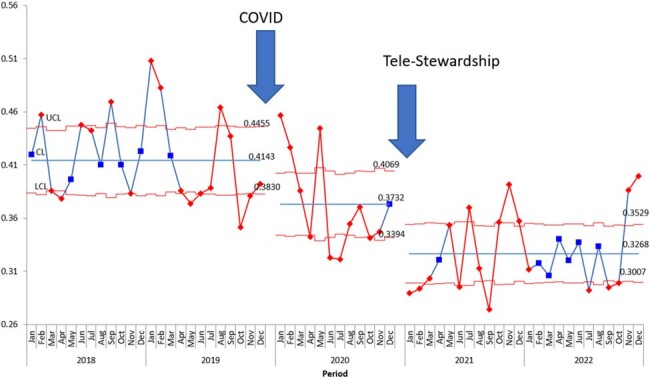
U chart of antimicrobial days of therapy per patient day

**Methods:**

This is a quasi-experimental, pre- post- analysis of a prospective audit and feedback PT-ASP across 23 medical centers throughout Intermountain Health in Utah. The pre-intervention group included patients < 18 years of age who had antibiotics at any of these 23 hospitals between January 1, 2018, and December 31, 2019. The post-intervention group included patients < 18 years of age who had antibiotics ordered between January 1, 2021, and December 31, 2022. Post-intervention patients were reviewed by a pediatric ASP pharmacist for a descriptive analysis of the daily census and interventions made. The primary outcome was antibiotic days of therapy/patient day (DOT) pre- and post-intervention. The secondary outcomes were to describe the types of interventions and the acceptance rates.

**Figure 2:**
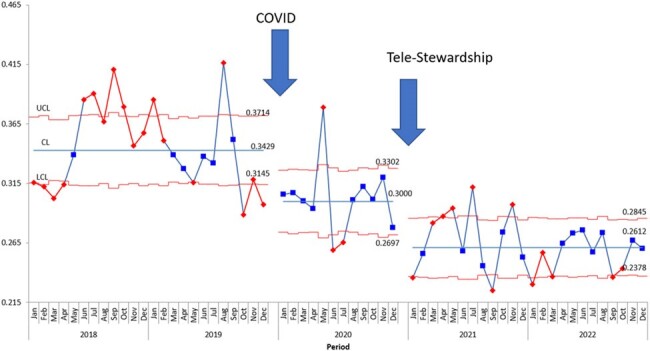
U chart of IV antimicrobial days of therapy per patient day

**Results:**

During the first two years of the PT-ASP implementation, 14,999 hospital census days with active antibiotic orders were reviewed. Compared to the pre-intervention period, there was a 21% decrease in total antimicrobial DOT per patient day and a 24% decrease in IV antimicrobial DOT per patient day during the intervention period. The census had a majority of NICU patients (51%). A total of 1,970 interventions were made on 1,287 unique patients, with 13% of the charts reviewed leading to an intervention. The acceptance rate of these interventions was 62%. The two most common types of interventions made were to change the antibiotic regimen and to discontinue antibiotics.

**Table 1:**
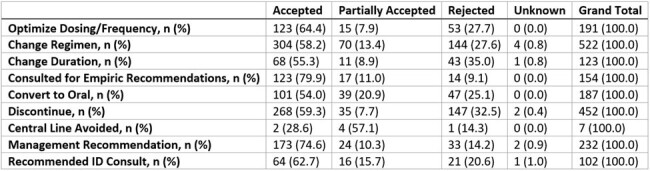
Pediatric tele-antimicrobial stewardship program intervention acceptance rates by type of intervention

**Conclusion:**

The PT-ASP made over 1,900 interventions leading to a decrease in both total antimicrobial DOT and IV antimicrobial DOT per patient day throughout 23 hospitals in an integrated health system. A PT-ASP is a strategy to steward pediatric beds outside of a stand-alone children’s hospital.

**Disclosures:**

**All Authors**: No reported disclosures

